# The new era shaped by environmental genome monitoring - symposium of the japanese environmental mutagen and genome society (JEMS), 2024

**DOI:** 10.1186/s41021-025-00327-x

**Published:** 2025-03-17

**Authors:** Hiroshi Honda, Takayoshi Suzuki, Masaaki Kitajima, Natsuko Ito Kondo, Kaede Miyata, Shunsuke Utsumi, Masami Yamada

**Affiliations:** 1 R&D Safety Science Research, Kao Corporation, Akabane, Ichikai-Machi, Haga-Gun, 321-3497 Tochigi, Japan; 2 Division of Genome Safety Science, National Institute of Health Sciences, Tonomachi, Kawasaki-ku, Kawasaki city, 210-9501 Kanagawa Japan; 3 Research Center for Water Environment Technology, School of Engineering, The University of Tokyo, Yayoi, Bunkyo-ku, Tokyo, 113-0032 Japan; 4 Bioaffiliationersity Division, National Institute for Environmental Studies, Onogawa, Tsukuba, 305-8506 Ibaraki Japan; 5 Faculty of Environmental Earth Science, Hokkaido University, Sapporo, 060-0810 Hokkaido Japan; 6 Department of Applied Chemistry, National Defense Academy, Hashirimizu, Yokosuka, 239-8686 Kanagawa Japan

**Keywords:** Environmental genomics, Metagenomics, eDNA, eRNA, Antibiotic resistance, Biodiversity, Public health

## Abstract

The symposium “The New Era Shaped by Environmental Genome Monitoring,” held in December 2024 by the Japanese Environmental Mutagen and Genome Society (JEMS), aimed to explore the interdisciplinary collaborations that are essential for the development of new scopes in environmental genome monitoring. This event highlighted the necessity of integrating mutagenicity research with ecological assessments to enhance public health and biodiversity conservation. Presentations focused on the evolving landscape of environmental genomics, including metagenomic analyses for antibiotic resistance, viral genomic surveillance in wastewater, and innovations in noninvasive biodiversity and stress monitoring through environmental DNA and RNA. This report summarizes the key discussions and presentations from the symposium, underscoring the critical role of environmental genome monitoring in shaping future safety research.

## Background

The traditional domain of mutagenicity and genotoxicity research has primarily focused on evaluating the adverse effects of chemicals on the genome, particularly in relation to carcinogenic risks. Meanwhile, recent advancements in next-generation sequencing (NGS) have utilized in not only human/animal genome analysis but also environmental genome analysis. Considering this trend, the symposium “The New Era Shaped by Environmental Genome Monitoring” was organized by Hiroshi Honda and Masami Yamada at the JEMS annual meeting 2024 in Okayama to explore how environmental genomics could be leveraged to address public health challenges and biodiversity conservation. The aim was to highlight the growing importance of interdisciplinary collaborations between researchers in environmental mutagenicity and ecology, thereby acquiring a new scope for the society.

## Main text

### Symposium Program

Chairs: Natsuko KONDO (Biodiversity Division, National Institute for Environmental Studies), Hiroshi HONDA (R&D Human Health Care, Kao Corporation).

1. Introduction
  Hiroshi HONDA (R&D Human Health Care, Kao Corporation).
2. Metagenomic Analysis of River Water for Bacterial Flora and Drug Resistance genes
  Presenters: Takayoshi SUZUKI (Division of Genome Safety Science, National Institute of Health Sciences), Kahoko NISHIKAWA (Faculty of Commerce, Chuo University).
3. Wastewater-based Epidemiology: Development and Social Implementation of Highly Sensitive Pathogen Genome Detection Technologies in the Environment.
  Presenter: Masaaki KITAJIMA (Research Center for Water Environment Technology, School of Engineering, The University of Tokyo).
4. Using Biodiversity Information through environmental DNA analysis and its challenges
  Presenter: Natsuko I. KONDO (National Institute for Environmental Studies).
5. The utility of Environmental RNA for Assessment of Biodiversity and Stress Response Analysis
  Presenter: Kaede MIYATA (Kao Corporation).
6. Perpetual, Reciprocal Interplay of Ecology and Evolution in the Wild
  Presenters: Shunsuke UTSUMI (Faculty of Environmental Earth Science, Hokkaido University), Fugen OHKUMA (Graduate School of Environmental Science, Hokkaido University), Naoki SHIMAMOTO (Graduate School of Environmental Science, Hokkaido University), Kinuyo YONEYA (Faculty of Agriculture, Kindai University).

### Meeting report

Takayoshi Suzuki (National Institute of Health Sciences) discussed the urgent issue of antibiotic-resistant bacteria, emphasizing that this has become a significant public health concern. He outlined how pollution, particularly from antibiotics, has led to an increase in resistant strains, making it crucial to monitor and detect these pathogens, and emphasized the importance of metagenomic analysis of river water as a key technology for investigating bacterial communities and understanding the distribution of drug resistance genes. He outlined two major challenges faced in this field: first, improving the efficiency of microbial recovery from environmental water samples, and second, accurately identifying the various forms of resistance genes [1]. He introduced the idea that biofilm or the fish gut can improve the efficiency of environmental DNA (eDNA) recovery from the water environment. He also highlighted innovative methods using long-read sequencing technologies and online search software to identify the bacteria and antimicrobial resistance genes.

Masaaki Kitajima (The University of Tokyo) presented on wastewater-based epidemiology (WBE) as an innovative approach for monitoring public health trends related to infectious diseases. He explained that WBE offers significant advantages, including the collection of continuous and unbiased data on pathogen prevalence in communities, regardless of individual symptoms or healthcare-seeking behaviors [2] He provided an overview of developed virus identification method including variant detection, which has been successfully implemented to monitor COVID-19 in the Tokyo 2020 Olympic and Paralympic Village [3]. His presentation illustrated the method’s applicability to other respiratory viruses, showcasing its potential for a broader public health surveillance framework. He emphasized that the application of WBE is crucial for establishing a multilayered infectious disease surveillance system, especially in the post-pandemic society.

Natsuko Kondo (The National Institute for Environmental Studies) focused on the application of eDNA analysis as a powerful tool for understanding species distribution in ecosystems. She explained that eDNA, which is derived from macroorganisms’ tissues and excretions, allows for the detection of species that are often difficult to observe through traditional ecological survey methods and presented examples of how eDNA is used in more interdisciplinary research [4]. She highlighted the challenges faced in eDNA analysis, such as the need for comprehensive reference databases to identify species accurately and the issue of pseudogenes that may lead to misestimations of biodiversity, and emphasized ongoing research efforts aimed at addressing these challenges, underscoring the significant potential of eDNA to inform conservation efforts and biodiversity assessments.

Kaede Miyata (Kao Corporation) introduced environmental RNA (eRNA) as an emerging tool for monitoring biodiversity [5] and assessing ecological responses to environmental stressors [6]. She showcased how eRNA can provide insights into the current state of ecosystems by detecting gene expression changes linked to environmental conditions. Her presentation included examples of various ecological surveys conducted using eRNA, demonstrating its effectiveness in accurately assessing the impacts of chemical pollutants on aquatic organisms. She discussed the advantages of using eRNA over traditional methods, emphasizing its potential for real-time monitoring of ecosystem health. She concluded by highlighting the importance of integrating eRNA technology into broader environmental management strategies to enhance sustainable practices.

Shunsuke Utsumi (Hokkaido University) discussed the dynamic interactions between ecology and evolution revealed by eDNA. He emphasized the critical importance of monitoring these interactions toward a better understanding of the changes and maintenance of biodiversity. He introduced the concept of eco-evolutionary feedback, which describes how evolutionary changes in species can influence ecological interactions, and vice versa [7]. He illustrated this concept with examples of rapid evolution occurring in response to urban environmental changes, discussing the implications for ecosystem management and biodiversity conservation [8]. His presentation underscored the need for integrating genetic and ecological data in research to develop a comprehensive understanding of these complex interactions.

## Conclusions

The symposium underscored the significant advancements in environmental genomics, proving essential for both public health and biodiversity conservation. Discussions revealed that metagenomic analyses have enhanced our ability to monitor antibiotic resistance, while WBE has provided a non-invasive method for tracking the trend of infectious disease prevalence in a community. The integration of eDNA and eRNA technologies is paving the way for improved biodiversity assessments and ecological monitoring. The environmental mutagenicity research could be extended to a comprehensive framework that integrates public health with environmental and genomic safety, highlighting the necessity for interdisciplinary collaboration in tackling complex global challenges. Future directions should discuss fostering collaboration and promoting technical transfer among researchers in genome safety research for holistic eco- and human health approaches.

## Data Availability

No datasets were generated or analysed during the current study.

## Abbreviations

eDNA: environmental DNA
eRNA: environmental RNA
NGS: next-generation sequencing
WBE: wastewater-based epidemiology
